# Supplementary material for: A highly active Burkholderia polyketoacyl-CoA thiolase for production of triacetic acid lactone
Source: Nat Commun. 2025 Dec 9;16:10990. doi: 10.1038/s41467-025-65946-y (PMC12689856; doi:10.1038/s41467-025-65946-y)
Supplement: Supplementary file 1 — Supplementary Information [file 41467_2025_65946_MOESM1_ESM.pdf]

A highly active *Burkholderia* polyketoacyl-CoA thiolase for production of triacetic acid lactone

Wang *et al.*

Supplementary Table 1. GenBank Accession numbers of proteins used in this study.

Protein	source	Accession number	Evolutionary distance
BktBct	*Cupriavidus taiwanensis*	WP_116336749.1	<0.004
BktBba	*Burkholderia ambifaria*	WP_012371925.1	0.014~0.023
BktBbr	*Burkholderia* sp. RF2-non_BP3	WP_059700748.1	0.014~0.023
BktBbb	*Burkholderia* sp. Bp8998	WP_124663072.1	0.014~0.023
BktBcn	*Cupriavidus necator*	WP_011615089.1	<0.004
BktBcl	*Cupriavidus* sp. L7L	WP_133094381.1	<0.004
BktBrs	*Rhodosalinus sediminis*	WP_115980015.1	>0.023
PhbAzr	*Zoogloea ramigera*	1DLV	n/a
2-PS	*Gerbera hybrida*	CAA86219.2	n/a

Supplementary Table 2. Results of Michaelis-Menten plot of all BktBs and 2-PS with only acetyl-CoA or malonyl-CoA. Data represent mean ± standard deviation (SD), n = 3 independent biological replicates, using separately prepared protein batches.

	BktBcn	BktBbb	BktBcl	BktBbr	BktBct	BktBba	2-PS
k_{cat} (min^{-1})	84.62±15.66	82.45±6.49	78.83±7.39	72.19±6.92	101.64±3.39	93.47±5.53	55.79±2.07
K_M (uM)	39.25 ± 20.43	43.34 ± 16.64	39.29 ± 6.64	23.99 ± 13.47	28.86 ± 2.80	27.93 ± 9.96	6.93 ± 1.34
k_{cat}/K_M (min^{-1} μM^{-1})	2.49 ± 0.97	2.1 ± 0.64	2.02 ± 0.37	3.01 ± 1.26	3.55 ± 0.33	3.63 ± 1.21	8.24 ± 1.49
R^2	0.96 ± 0.02	0.93 ± 0.04	0.98 ± 0.01	0.89 ± 0.04	0.99 ± 0.01	0.98 ± 0.02	0.94 ± 0.01

Source data are provided as a Source Data file.

Supplementary Table 3. Data collection and refinement statistics.
Values in parentheses are for the highest-resolution shell.

	**BktB *apo* enzyme**	**BktB-butyryl-CoA**	**BktB-acetyl-CoA**	**BktB-acetoacetyl-CoA**
**Data collection**				
Space group	P 43 21 2	P 43 21 2	P 43 21 2	P 43 21 2
Unit-cell parameters (Å)	a=130.47 b=130.47 and c=122.08	a=130.32 b=130.32 and c=120.70	a=130.29 b=130.29 and c=121.80	a=130.77 b=130.77 and c=121.59
Resolution range (Å)	39.09 - 2.2 (2.27 - 2.2)	38.44 - 2.1 (2.17 - 2.1)	43.09 - 2.23 (2.31 - 2.23)	46.24 - 2.45 (2.53 - 2.45)
R_{merge} (%)	0.133 (3.05)	0.099 (2.47)	0.155 (3.31)	0.120 (1.345)
$I/\sigma I$	18.6 (1.5)	17.8 (1.3)	15.1 (0.8)	22.5 (2.2)
Completeness (%)	99.8 (98.9)	99.8 (99.0)	99.9 (99.9)	100 (100)
Redundancy	27.4 (27.2)	18.2 (18.4)	13.1 (12.9)	14.4 (15.0)
$CC_{1/2}$	0.999 (0.673)	0.999 (0.558)	0.992 (0.309)	0.999 (0.773)
**Refinement**				
Resolution range (Å)	39.09 - 2.2 (2.27 - 2.2)	38.44 - 2.1 (2.17 - 2.1)	43.09 - 2.23 (2.31 - 2.23)	46.24 - 2.45 (2.53 - 2.45)
Reflections used in refinement	50004 (4130)	60962 (6008)	51555 (5049)	39324 (3862)
R_{work}	0.174 (0.239)	0.177 (0.283)	0.196 (0.381)	0.191 (0.250)
R_{free}	0.216 (0.286)	0.194 (0.288)	0.227 (0.446)	0.226 (0.288)
No. atoms				
Proteins	5719	5736	5724	5724
Ligands/ion	0	168	165	108
Water	137	309	178	237
RMS from ideal geometry				
Bond lengths (Å)	0.012	0.009	0.003	0.003
Bond angles (°)	1.26	0.88	0.62	0.52
Average B-factor	62.8	65.3	64.5	66.1
Macromolecules	63.06	64.9	63.5	65.9
Ligands	n/a	96.3	133.1	92.8
Solvent	54.1	63.1	56.7	57.9
PDB ID	9BWK	9BWL	9BWO	9BWP

Supplementary Table 4. Mutants of BktBs.

Code	ID	Mutants and annotation
JBx_257938	Mutant-1	BktBcn_T17D_loop
JBx_257940	Mutant-2	BktBcn_VAPAELGALVVREALARAQ25-43FSPTDLGAKVVSEVLSRAN_helix
JBx_257942	Mutant-3	BktBcn_D48A_helix
JBx_257944	Mutant-4	BktBcn_NVIQ56-59HVVN_loop_close to active pocket
JBx_257946	Mutant-5	BktBcn_RDMYLGRVAAVNGGVTINA63-81KDMYLARVAAINGGVAQHT_helix
JBx_257948	Mutant-6	BktBcn_LLGDT104-109MLGDA_loop
JBx_257950	Mutant-7	BktBcn_AES117-119SEN_sheet
JBx_257952	Mutant-8	BktBcn_LAPAARWGARMGDAG126-140TVPSARFGQRMGDAK_helix
JBx_257954	Mutant-9	BktBcn_HR154-155QT_loop
JBx_257956	Mutant-10	BktBcn_KEYD167-170RKYG_helix
JBx_257958	Mutant-11	BktBcn_SRAQQDEAALESHRRASAAIKAGY172-195TRDAQDALALESHRRAARAIAEGR_helix_close to coa
JBx_257960	Mutant-12	BktBcn_VPVVSKGRKGDVT201-213LPISIRTKKGEVA_helix
JBx_257962	Mutant-13	BktBcn_TI225-226GP_helix_close to coa
JBx_257964	Mutant-14	BktBcn_M229F_helix_close to coa
JBx_257966	Mutant-15	BktBcn_RPVFVKENGTVTAGNASGL233-251KPVFVKEDGTVTAGNASGI_loop_enfolding coa
JBx_257968	Mutant-16	BktBcn_L251I_loop_partial coa channel
JBx_257970	Mutant-17	BktBcn_VMMERAEAERRGL259-271LMMSADAARAQGV_sheet-helix
JBx_257972	Mutant-18	BktBcn_SYGHAGVDPKAM279-290AYAHAGVDPAYM_sheet-helix_partial active pocket
JBx_257974	Mutant-19	BktBcn_KIALERAGLQVS299-310QKALERAGLKIT_helix-helix
JBx_257976	Mutant-20	BktBcn_KA330-331QE_helix
JBx_257978	Mutant-21	BktBcn_HELNRVQ365-371YELKRIG_helix
JBx_257980		BktBcn_I58V
JBx_257982		BktBcn_L251I
JBx_257984		BktBbr_C95S
JBx_257986		BktBbr_H162A
JBx_257988		BktBbr_H189A
JBx_257990		BktBbr_H224A
JBx_257992		BktBbr_R226A
JBx_257994		BktBbr_G251A
JBx_257996		BktBbr_S254A
JBx_257998		BktBbr_S254C
JBx_258000		BktBbr_G255A
JBx_258002		BktBbr_I256A
JBx_258004		BktBbr_H355A
JBx_258006		BktBbr_G387A

Supplementary Table 5. Strains and plasmids used in this study.

Strain/Plasmid	Genotype	Source	Code
**Strain**			
BW25113	*lacI*$^{+}$ *rrnB*$_{T14}$ Δ*lacZ*$_{WJ16}$ *hsdR*514 Δ*araBAD*$_{AH33}$ Δ*rhaBAD*$_{LD78}$ *rph-1*Δ*(araB–D)567* Δ*(rhaD–B)568* Δ*lacZ4787*(::*rrnB-3*) *hsdR514 rph-1*	Baba *et al.* [1]	
BL21(DE3)	B F^{-} *ompT gal dcm lon hsdS*$_{B}$($r_{B}^{-}m_{B}^{-}$) λ(DE3 [*lacI lacUV5-T7p07 ind1 sam7 nin5*]) [*malB*$^{+}$]$_{K-12}$(λ^{S})	New England Biolabs	
JBEI-3695	BW25113 Δ*adhE*::FRT Δ*ldhA*::FRT Δ*frdBC*::FRT Δ*pta*::FRT	This study	
**Plasmid**			
pET28a(+)-TEV	ColE1 *ori*; P_{T7}	Genscript	
pET28_his6_TEV_bktBct	ColE1 *ori*; Kan^{R}; P_{T7}: *bktBct* with N-terminal 6x his-tag and TEV protease site	This study	JBx_257932
pET28_his6_TEV_bktBba	ColE1 *ori*; Kan^{R}; P_{T7}: *bktBba* with N-terminal 6x his-tag and TEV protease site	This study	JBx_257934
pET28_his6_TEV_bktBbr	ColE1 *ori*; Kan^{R}; P_{T7}: *bktBbr* with N-terminal 6x his-tag and TEV protease site	This study	JBx_257930
pET28_his6_TEV_bktBbb	ColE1 *ori*; Kan^{R}; P_{T7}: *bktBbb* with N-terminal 6x his-tag and TEV protease site	This study	JBx_257926
pET28_his6_TEV_bktBcn	ColE1 *ori*; Kan^{R}; P_{T7}: *bktBcn* with N-terminal 6x his-tag and TEV protease site	This study	JBx_257922
pET28_his6_TEV_bktBcl	ColE1 *ori*; Kan^{R}; P_{T7}: *bktBcl* with N-terminal 6x his-tag and TEV protease site	This study	JBx_257928
pET28_his6_TEV_bktBrs	ColE1 *ori*; Kan^{R}; P_{T7}: *bktBrs* with N-terminal 6x his-tag and TEV protease site	This study	JBx_257924
pBbA5a-RFP	p15A *ori*; Amp^{R}; P_{lacUV5}: *RFP*	Lee *et al.* [2]	
pBbS5a-RFP	SC101 *ori*; Amp^{R}; P_{lacUV5}: *RFP*	Lee *et al.* [2]	
pBbE5a-RFP	ColE1 *ori*; Amp^{R}; P_{lacUV5}: *RFP*	Lee *et al.* [2]	
pBbE1a-RFP	ColE1 *ori*; Amp^{R}; P_{Trc}: *RFP*	Lee *et al.* [2]	
pBbA5a-bktBct	p15A *ori*; Amp^{R}; P_{lacUV5}: *bktBct*	This study	JBx_148831
pBbA5a-bktBba	p15A *ori*; Amp^{R}; P_{lacUV5}: *bktBba*	This study	JBx_148832
pBbA5a-bktBbr	p15A *ori*; Amp^{R}; P_{lacUV5}: *bktBbr*	This study	JBx_148833
pBbA5a-bktBbb	p15A *ori*; Amp^{R}; P_{lacUV5}: *bktBbb*	This study	JBx_148834
pBbA5a-bktBcn	p15A *ori*; Amp^{R}; P_{lacUV5}: *bktBcn*	This study	JBx_148835
pBbA5a-bktBcl	p15A *ori*; Amp^{R}; P_{lacUV5}: *bktBcl*	This study	JBx_148836
pBbA5a-bktBrs	p15A *ori*; Amp^{R}; P_{lacUV5}: *bktBrs*	This study	JBx_148837
pBbA5a-2PS	p15A *ori*; Amp^{R}; P_{lacUV5}: *2-PS*	This study	JBx_227414
pBbS5a-bktBbr	SC101 *ori*; Amp^{R}; P_{lacUV5}: *bktBbr*	This study	JBx_228660
pBbE5a-bktBbr	ColE1 *ori*; Amp^{R}; P_{lacUV5}: *bktBbr*	This study	JBx_228661
pBbE1a-bktBbr	ColE1 *ori*; Amp^{R}; P_{trc}: *bktBbr*	This study	JBx_228662

Supplementary Table 6. LC-MS method and parameters for TAL identification and quantification.

	Time (min)	A (%)	B (%)	Flow rate (mL min^{-1})
General chromatography	0	80	20	0.42
	6.5	28	72	0.42
	7.8	5	95	0.42
	8.8	5	95	0.42
	9	80	20	0.42
	11.2	80	20	0.42
High-throughput chromatography	0	80	20	0.42
	2.8	5	95	0.42
	4.5	5	95	0.42
	4.6	80	20	0.60
	6.0	80	20	0.60
Column temperature (°C)	40			
DAD (nm)	254, 298			
MS scan $[M-H]^-$	*m/z* 100-200			
SIM-1, *m/z*	126.12	TAL		
SIM-2, *m/z*	170.21	Internal standard, (5*S*,6*S*)-6-isopropyl-5-methyldihydro-2*H*-pyran-2,4(3*H*)-dione		

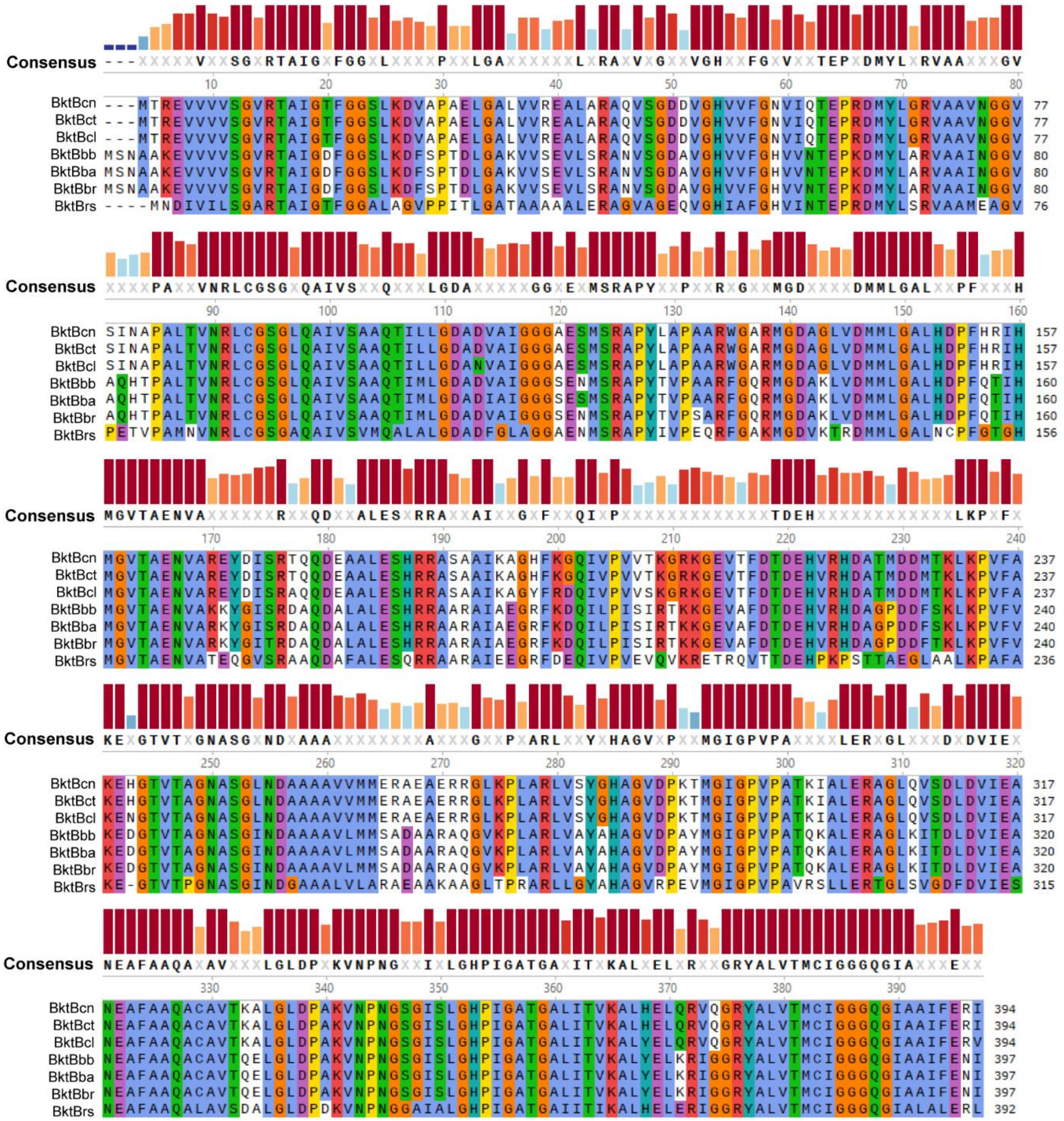

Supplementary Fig. 1. Complete protein sequence alignment of BktBs. Seven selected BktBs were aligned by MAFFT. The consensus amino acid residues are highlighted in different colors and shown in the Consensus row. The bar graph shown above each sequence alignment represents the identity % of amino acids at different positions.

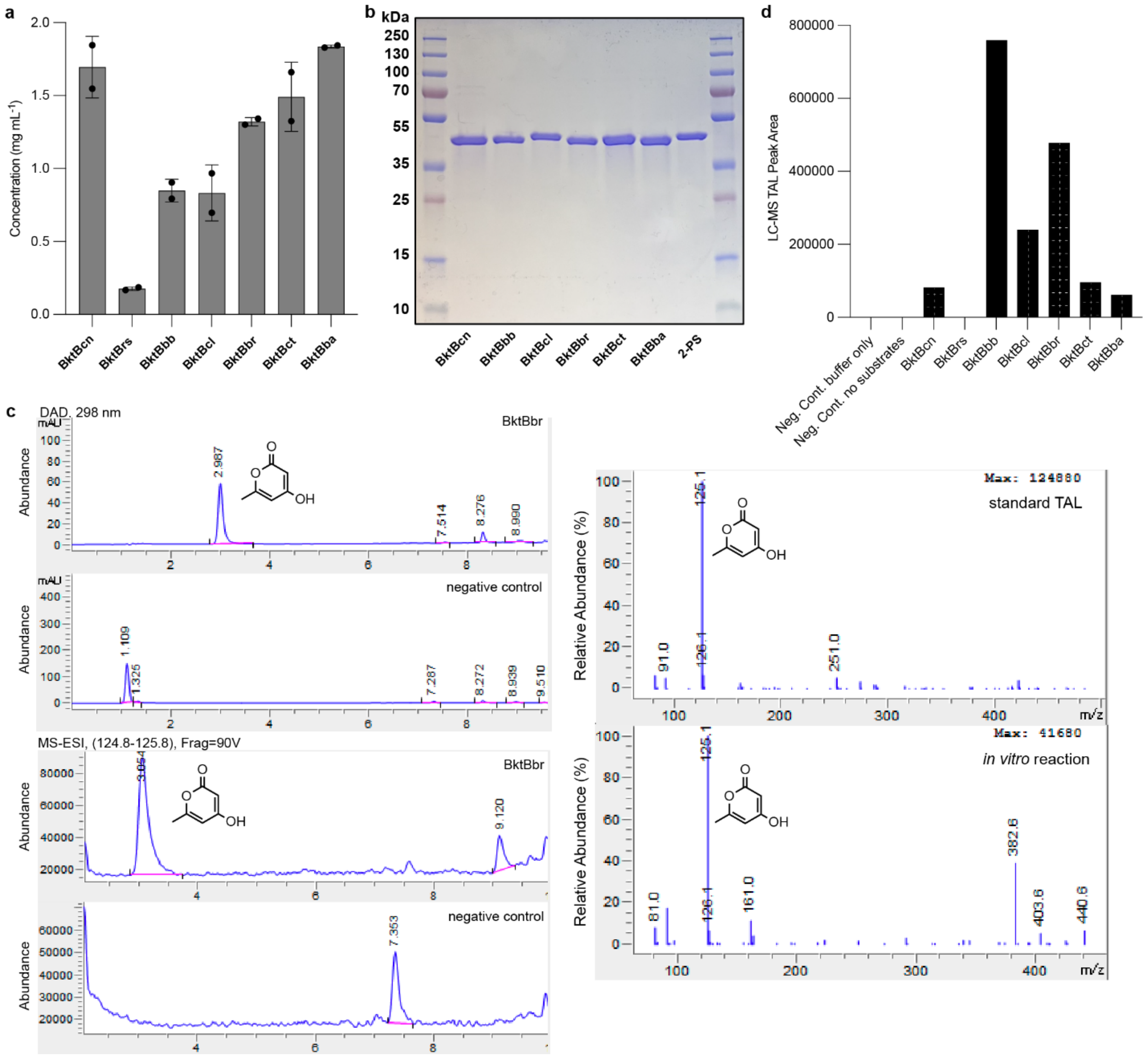

Supplementary Fig. 2. Purified proteins and LC-MS of *in vitro* TAL formation. a) Protein concentrations, measured by Bradford assay, of BktBs produced using *E. coli* BL21(DE3) and purified using nickel resin. b) SDS-PAGE gel result of purified proteins used in this study. 5.5 µg of proteins was loaded into each lane. SDS-PAGE gel (Bio-Rad, USA) was precast to 4~20 %, run at 140 V for 1 h. c) LC-MS analysis of TAL and its identification using mass spectrometry. TAL eluted at 2.987 min. Mass spectrum of pure TAL standard and *in vitro* reaction of TAL production are shown on the right. *m/z* of 125.1 is TAL. d) Amounts of TAL produced by various BktBs and quantified by LC-MS. 1 mM acetyl-CoA was incubated with BktBs at 25 °C for 1 h. Source data are provided as a Source Data file.

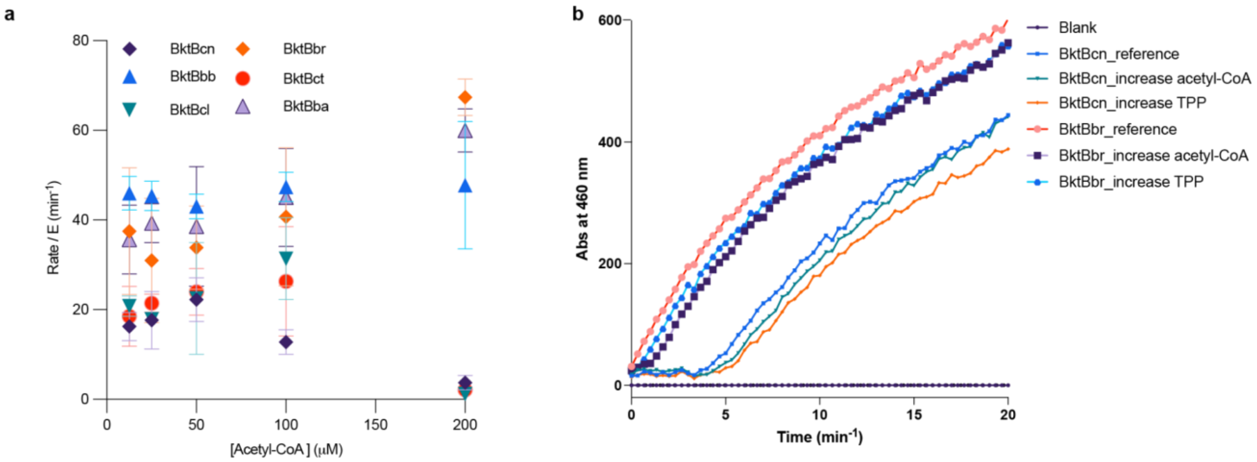

c $$v = k_1[AcAcCoA]^{m1}[AcCoA]^{m2} - k_2[AcAcCoA]^{n1}[CoA]^{n2}$$

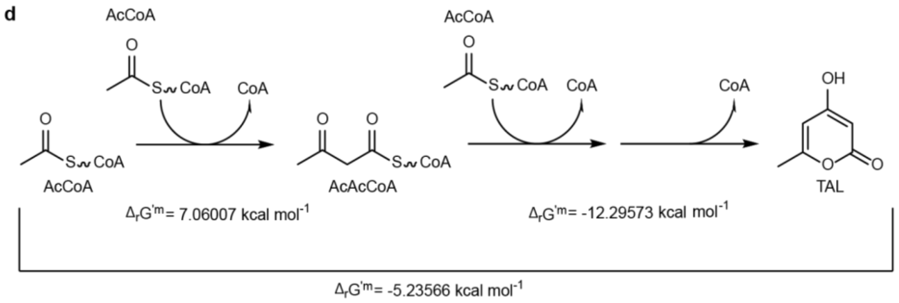

Supplementary Fig. 3. Optimization of kinetic assay and the Michaelis-Menten plot of all BktBs. a) Michaelis-Menten plot of the wild-type BktBs. The enzymes were assayed using the same concentration of acetyl-CoA at 0.2 mM and various concentrations of acetoacetyl-CoA: 12.5, 25, 50, 100, and 200 μM. The data are from three biological replicates (error bars indicate mean values + standard deviation). b) Optimization of kinetics assay. Compared with the reference BktB assay performed with 0.2 mM acetyl-CoA and 0.2 mM acetoacetyl-CoA, acetyl-CoA was increased to 2 mM to enhance the TAL formation (indicated by "_increase acetyl-CoA" in label). As TPP was the limiting component in the α-KGDH assay, the TPP concentration was increased from 0.4 mM to 4 mM (indicated by "_increase TPP" in labels). All experiments have three replicates. The data were analyzed by Graphpad. c) Hypothetical rate of TAL formation and thiolysis of acetoacetyl-CoA. During the initial phase of the reaction, in the presence of a small quantity of CoA, the substrate is essential for acetoacetyl-CoA thiolysis, which might be generated through the hydrolysis of acetoacetyl-CoA and acetyl-CoA, the thiolysis process of acetoacetyl-CoA can initiate. Consequently, the overall rate of TAL formation (and hence the overall rate of free CoA released to react in the coupled α-KGDH assay), denoted as v, can be approximated as the synthesis rate of TAL with rate constant k_1 subtracted by the rate of thiolysis with rate constant k_2. *AcAcCoA* is acetoacetyl-CoA, *AcCoA* is acetyl-CoA. d) Thermodynamics of the BktB reactions. Standard Gibbs free energy change of reaction $\Delta_rG'^{\circ}$ was first computed from values of standard Gibbs free energy of formation ($\Delta_fG'^{\circ}$) for compounds involved in the reaction. Values for $\Delta_fG'^{\circ}$ were obtained from the MetaCyc database (https://metacyc.org/), which estimates $\Delta_fG'^{\circ}$ using the group contribution method under standard conditions (298.15 K, pH 7.3 and an ionic strength of 0.25)[3,4].With the unit of kcal mol^{-1}, $\Delta_fG'^{\circ}$ of CoA is -493.56613, acetyl-CoA is -506.8639, acetoacetyl-CoA is -513.1016, and TAL is -41.03646. Because $\Delta_rG'^{\circ}$ is based on 1 M standard reactant concentration, which is not physiologically appropriate, $\Delta_rG'^{m}$ is then calculated through the equation $\Delta_rG'=\Delta_rG'^{\circ} + RT\ln Q_r$ (Q_r is reaction quotient) and ultimately applied as the thermodynamics of the reactions, in which all reactant concentrations are set as physiologically appropriate 1 mM[5]. Source Data are provided as a Source Data file.

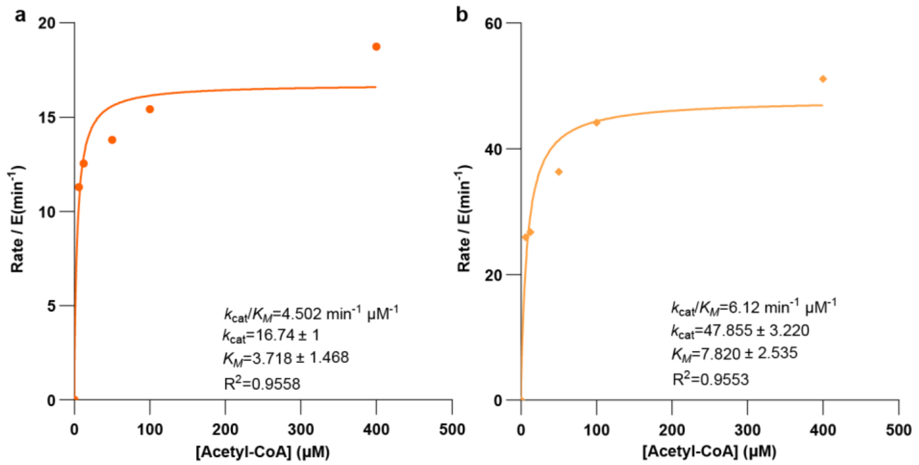

Supplementary Fig. 4. Michaelis-Menten plots of BktBcn and BktBbr with LC-MS measurement of TAL formation. a) BktBcn, b) BktBbr. Time-course assays were performed using a fixed concentration of acetoacetyl-CoA (400 µM) and varying acetyl-CoA concentrations (0, 6.25, 12.5, 50, 100, and 400 µM) to isolate the productive condensation step. TAL formation was quantified by high-sensitivity LC-MS. For BktBcn, initial velocities were calculated from time points spanning 5–45 min, during the linear phase of TAL accumulation. Although a brief lag precedes detection, this reflects instrument sensitivity and intermediate kinetics rather than absence of catalysis. The acetyl-CoA concentrations span below, near, and above the K_M values for both enzymes (3.7 µM for BktBcn and 7.8 µM for BktBbr). While additional lower concentrations could further refine the curve, those below 5 µM produced TAL levels below the LC-MS detection limit (1–3 µM). The k_{cat}, K_M, and R^2 values are indicated as mean values + standard deviations in each panel. Error bars are omitted for clarity. The data are from three biological replicates for a–b). Source data are provided as a Source Data file.

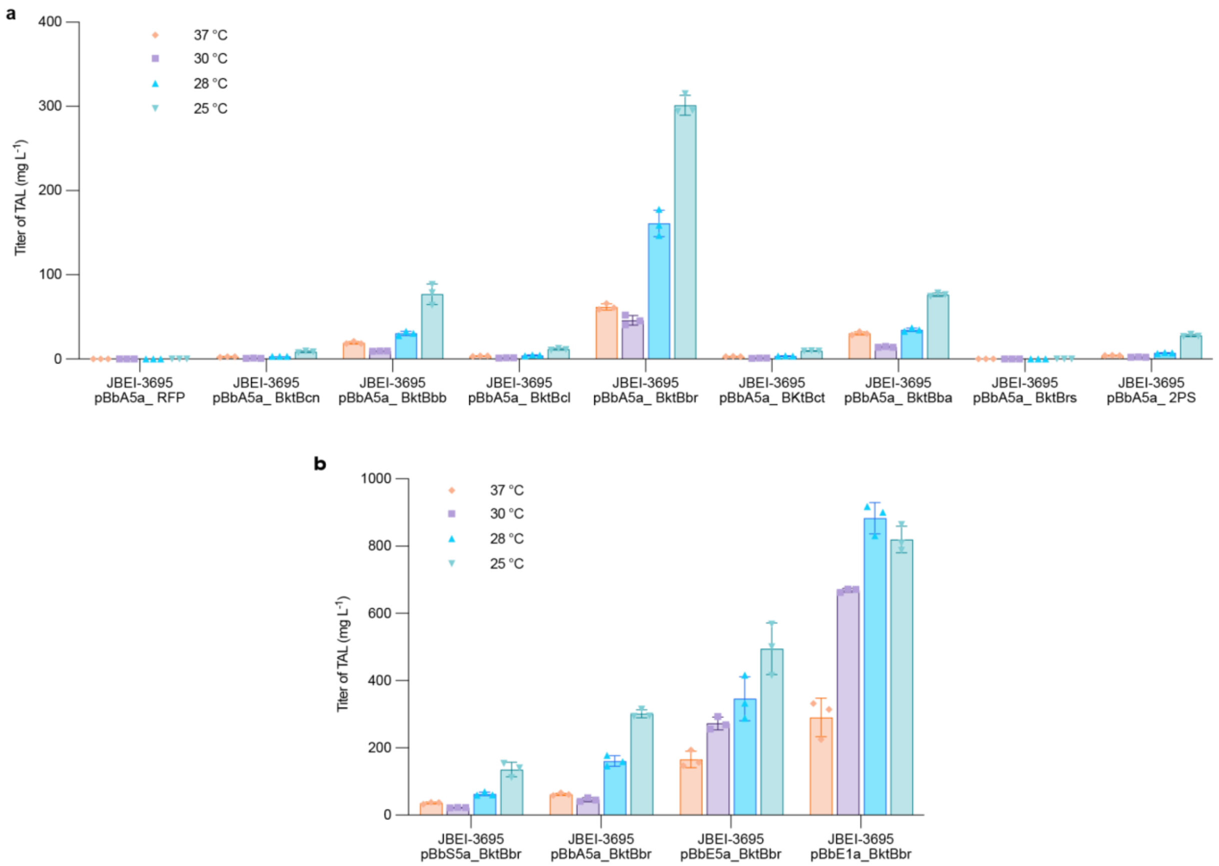

Supplementary Fig. 5. *In vivo* TAL production by *E. coli* strains under different temperatures. a) TAL titers of strains expressing different BktBs and 2-PS in 24-well plates. b) TAL titers of strains expressing *bktBbr* on Biobrick vectors with different copy numbers and promoter strengths**.** The host strain was JBEI-3695 (BW25113 Δ*adhE* Δ*ldhA* Δ*frdBC* Δ*pta*), and strains were cultivated with glycerol as the carbon source in 24-well plates. The data are from three biological replicates (error bars indicate mean values + standard deviation) for a–b). Source data are provided as a Source Data file.

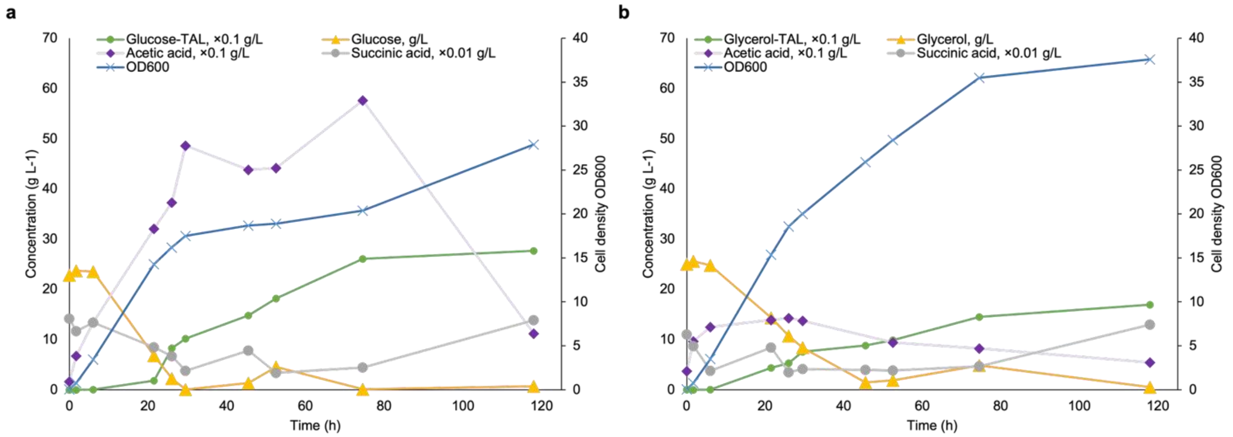

Supplementary Fig. 6. Analysis of 1-L fed-batch production studies with *E. coli* JBEI-3695. Samples were collected at different time points, and the glucose, glycerol, and metabolites concentrations were analyzed by HPLC. a) Growth and production with glucose in the medium. b) Growth and production with glycerol in the medium. The y axis is adjusted to be the same between a) and b). Source data are provided as a Source Data file.

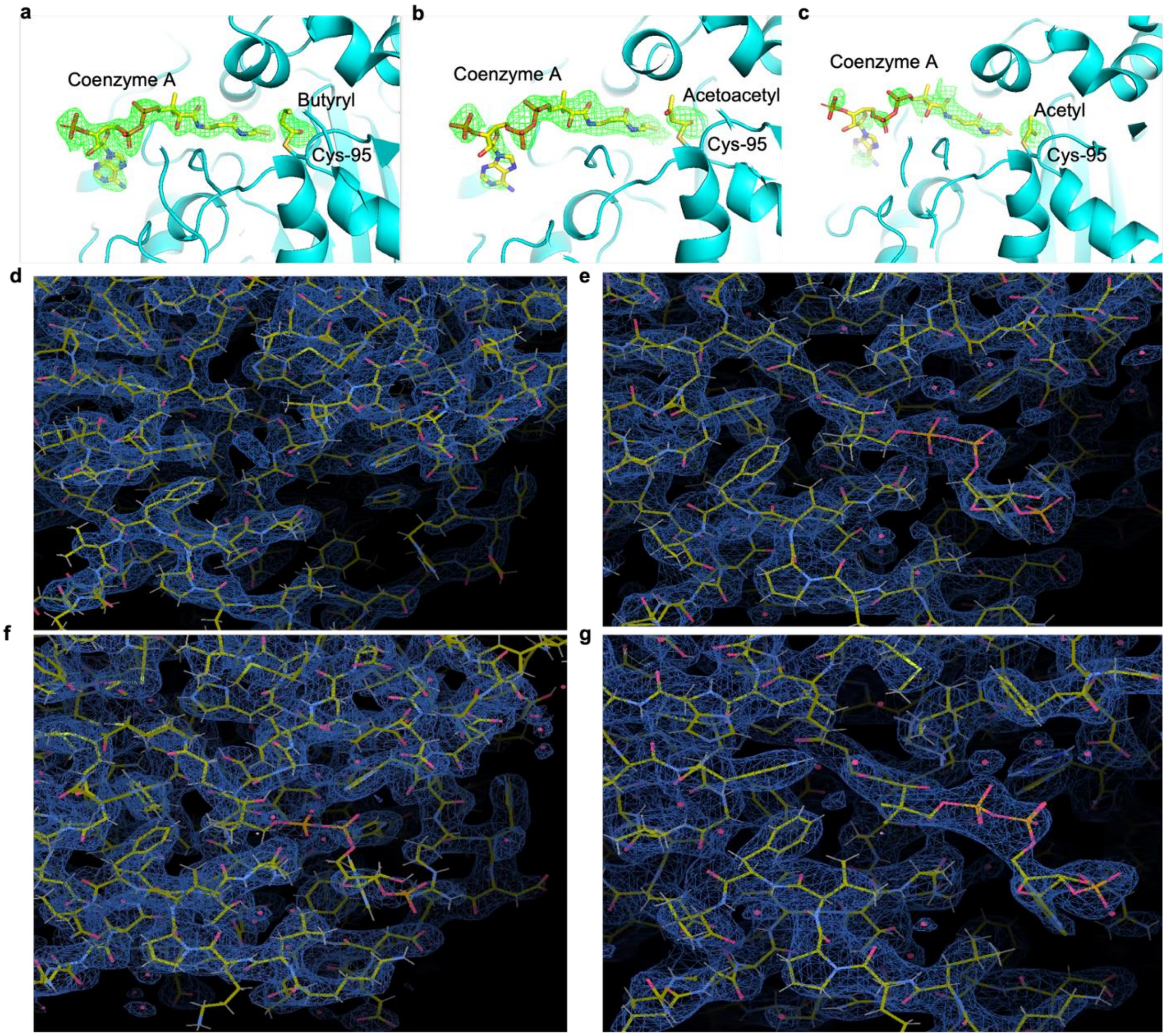

Supplementary Fig. 7. Binding modes of acyl-CoA substrates in the active site of BktBbr. a) Butyryl-CoA, b) Acetoacetyl-CoA, and c) Acetyl-CoA are shown bound in the substrate tunnel of BktBbr, with the Coenzyme A moiety extending outward and the acyl group positioned near the catalytic residue Cys95. Stick models represent the refined ligand coordinates. The green mesh corresponds to the mFo–DFc omit electron density, calculated by omitting the entire ligand during map refinement to avoid model bias. Density is contoured at 3.0 σ, clearly supporting the presence and positioning of each ligand and its covalent interaction with Cys95. d–f) 2mFo-DFc electron density map contoured at 1.0 sigma level showing the residues around the coA active for d) BktB-apo enzyme, e) Butyryl, f) Acetyl and g) Acetoacetyl.

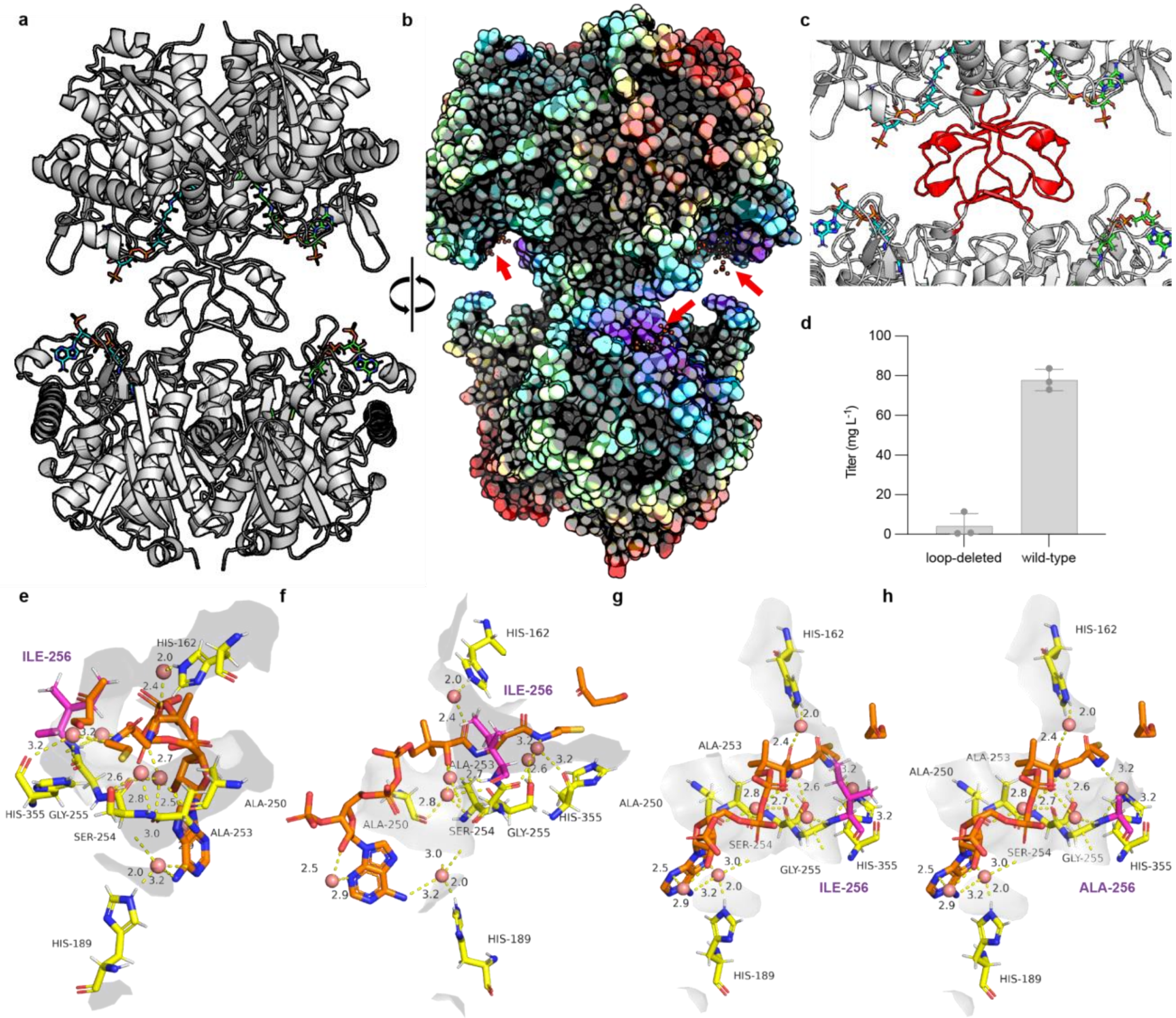

Supplementary Fig. 8. Tetrameric structure features of BktBbr. a) Tetrameric structure of BktBbr with acetyl-CoA (PDB ID: 9BWO). BktBbr is colored gray. The carbon of Acetyl-CoA chain is colored in orange color. The blue color represents the nitrogen, and the red color represents the oxygen. b) Surface charges of the tetrameric BktBbr structures with acetyl-CoA. Acetyl-CoA visible is highlighted with red arrows. From red color to purple color, the surface charges are from positive (red), to neutral (gray), to negative (purple). c) The tetrameric loop regions of BktBbr. The loop region is highlighted in red color. d) TAL production of BktBbr mutant with the entire loop region deleted. The mutant is YTVPSARFGQRMGDAKLVD124-149GSG. The production study was performed in a 24-well plate with the corresponding plasmid transformed in *E. coli* BL21(DE3). The data are from three biological replicates (error bars indicate mean values + standard deviation). e-g) The CoA binding tunnel viewed at different orientations. I256 is shown as ILE-256. ILE-256 is in purple color. h) The CoA binding tunnel viewed at the same orientation with g, where I256 was mutated to A256. A256 is shown as ALA-256, in purple color. The binding tunnel of the CoA was expanded, and the space of the tunnel with A256 was significantly larger than with I256. Source data are provided as a Source Data file.

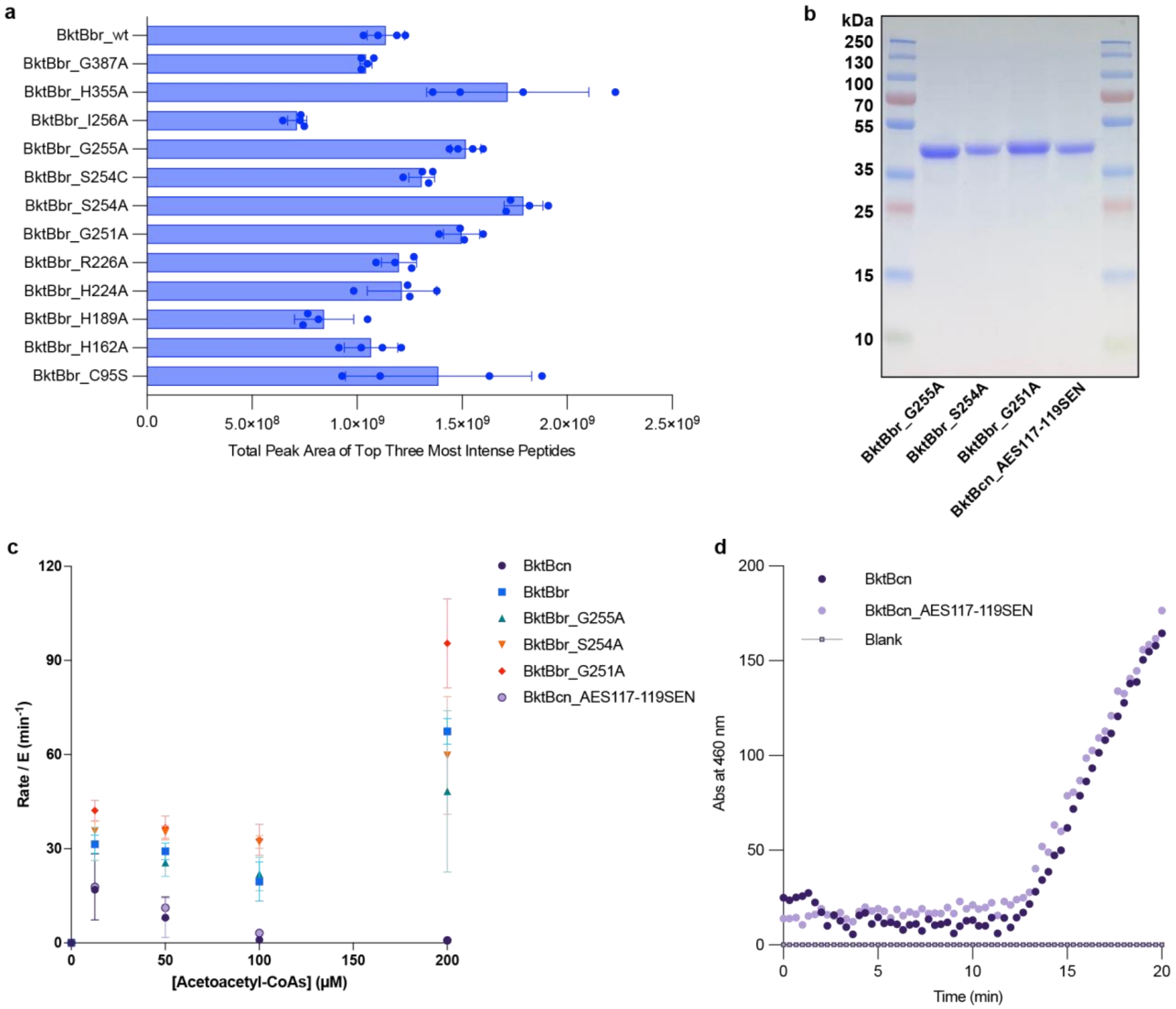

Supplementary Fig. 9. Mutagenesis study of BktBs analyzed *in vivo* and *in vitro*. a) Proteomics result of the BktBbr mutant expression level in *E. coli* BL21(DE3) after production studies. b) SDS-PAGE result of the purified selected BktBbr mutants. c) Michaelis-Menten plot of BktB mutants. The reaction contains 0.2 mM acetyl-CoA, and the gradient concentrations of acetoacetyl-CoA are 12.5, 25, 50, 100, and 200 µM acetoacetyl-CoA. d) Comparison of kinetics assay of BktBcn and BktBcn_AES117-119SEN. The reaction was performed under 200 µM acetyl-CoA and 200 µM acetoacetyl-CoA. No difference was observed for the lag phase. The data were analyzed and presented by Prism Graphpad. The data are from three biological replicates (error bars indicate mean values + standard deviation). Source data are provided as a Source Data file.

Supplementary references

1. Baba, T. *et al.* Construction of Escherichia coli K-12 in-frame, single-gene knockout mutants: the Keio collection. *Mol. Syst. Biol.* **2**, 2006.0008 (2006).
2. Lee, T. S. *et al.* BglBrick vectors and datasheets: A synthetic biology platform for gene expression. *J. Biol. Eng.* **5**, 12 (2011).
3. Jankowski, M. D., Henry, C. S., Broadbelt, L. J. & Hatzimanikatis, V. Group contribution method for thermodynamic analysis of complex metabolic networks. *Biophys. J.* **95**, 1487–1499 (2008).
4. Caspi, R. *et al.* The MetaCyc database of metabolic pathways and enzymes and the BioCyc collection of pathway/genome databases. *Nucleic Acids Res.* **44**, D471-480 (2016).
5. Flamholz, A., Noor, E., Bar-Even, A. & Milo, R. eQuilibrator--the biochemical thermodynamics calculator. *Nucleic Acids Res.* **40**, D770-775 (2012).
